# Expanding Access to 3D Technology in Plastic Surgery of the Breast: Validation of the Iphone X 3D Camera Against the Vectra M1

**DOI:** 10.1093/asjof/ojad027.012

**Published:** 2023-04-14

**Authors:** Hayeem Rudy, Yi-Hsueh Lu, Oren Tepper, Katie Weichman

**Affiliations:** Montefiore Medical Center, Bronx, NY; Montefiore Medical Center, Bronx, NY; Montefiore Medical Center, Bronx, NY; NYU Langone Medical Center, New York, NY

## Abstract

**Goals/Purpose:**

The iPhone X is the first mainstream smartphone to be released that contains a high-fidelity 3D scanner and is already in the hands of tens of millions of individuals in the United States. At the present time, the vast majority of 3D analysis of the breast necessitates ownership of expensive cost-prohibitive camera and software packages. The most popular of these is the Canfield Vectra M1 system. In an effort to expand access to 3D technology in plastic surgery of the breast, the purpose of this study was to compare the accuracy of the iPhone X 3D scanner against the Canfield Vectra M1 in obtaining 3D scans of the breast.

**Methods/Technique:**

Twenty breasts (n=20) in patients undergoing breast reconstructive surgery were 3D photographed using both the iPhone X and the Canfield Vectra M1. The accuracy of the iPhone 3D image was compared with the Vectra image using color map analysis and surface distances across the 3D model between key anatomical landmarks. These included distances between sternal notch to nipple (SN-N), mid-chest to nipple (M-N), nipple to mid-inframammary fold (N-IMF), and inframammary fold width (IMF).

**Results/Complications:**

When comparing absolute differences in distances between key anatomical landmarks, the average discrepancy in measurements between iPhone and Vectra image pairs were the following: SN-N: 0.84mm, M-N: 0.70mm, N-IMF 0.81mm, and IMF 0.91mm. Colormap analysis demonstrated an average root mean square error of 1.85mm, with a mean of 0.57mm and standard deviation of ±1.88mm.

**Conclusion:**

The iPhone X is capable of capturing 3D photographs of the breast with an average discrepancy of approximately 1.0 to 2.0 millimeters as determined by landmark to landmark surface distance analysis and color map analysis. These data demonstrate that the 3D scans obtained with the iPhone may be useful for determining nipple position, measuring the breast footprint, choosing implants, and performing other functions using 3D technology are typically performed using the more expensive systems. These are early data and additional studies should compare volumetric analysis and clinical outcomes between using the iPhone and more established 3D technology systems in breast reconstructive surgery.

